# Current trends and hotspots in drug-resistant epilepsy research: Insights from a bibliometric analysis

**DOI:** 10.3389/fneur.2022.1023832

**Published:** 2022-11-03

**Authors:** Xiao-Jun Ni, Han Zhong, Yang-Xi Liu, Hou-Wen Lin, Zhi-Chun Gu

**Affiliations:** Department of Pharmacy, Ren Ji Hospital, Shanghai Jiao Tong University School of Medicine, Shanghai, China

**Keywords:** drug-resistant epilepsy, bibliometric analysis, visualization, hotspots, VOSviewer

## Abstract

**Background:**

Drug-resistance is a significant clinical issue in persons with epilepsy. In the past few years, many studies have been published investigating the management of drug-resistant epilepsy (DRE); however, no systematic and quantitative evaluation of this research has been performed. Therefore, a bibliometric analysis was conducted to demonstrate the current status of DRE research and to reflect the trends and hotspots within the field.

**Methods:**

We retrieved publications on DRE published between 2011 and 2021 from the Science Citation Index Expanded of the Web of Science Core Collection. All articles related to DRE were included in this study. VOSviewer, R software, and CiteSpace were used to perform bibliometric research.

**Results:**

A total of 3,088 original articles were included in this study. The number of publications on DRE has continued to increase over the past 11 years. The USA published the most papers with the highest number of citations and H-index. The National Institutes of Health and the University of Toronto were the most prolific funding agency and affiliation, respectively. *Epilepsy* & *Behavior* and *Epilepsia* ranked first as the most prolific and co-cited journals, respectively. The keywords “cannabidiol”, “neuromodulation”, “seeg” and “perampanel” revealed recent research hotspots. The top 100 most cited papers were classified into eight main topics, of which pharmacotherapy, disease mechanisms/pathophysiology, and neuromodulation were the three most important topics.

**Conclusions:**

This analysis of bibliometric data demonstrated that DRE has always been a topical area of research. The mechanisms of epilepsy and therapies have been the focus of DRE research, and innovative antiseizure medications and surgical approaches are fast-developing research trends.

## Introduction

Epilepsy is one of the most prevalent and disabling chronic neurologic diseases, with 60 million patients globally and 125,000 deaths per annum (1, 2). Although antiseizure medications (ASMs) are effective at preventing seizures in most cases of epilepsy, 30–40% of patients develop resistance to drugs. This indicates that seizures persist despite the daily use of at least two syndrome-adapted ASMs (3). The failure of a secondary ASM to prevent seizures provides a basis for the consideration of alternative treatments, including surgery, neurostimulation, ketogenic diets, biofeedback, and gene therapy (4). Patients with drug-resistant epilepsy (DRE) contribute disproportionally to the global burden of epilepsy as they are associated with higher risks of morbidity and mortality, psychosocial and behavioral difficulties, cumulative brain atrophy, and progressive impairment of cognitive function (5).

The management of DRE has become one of the biggest challenges in clinical epilepsy practice. In the current era of the emergence of novel medication and technology, quantitative analyses are needed to provide an overview of incremental improvement, helping researchers capture the status, trends, and hotspots of DRE research.

Bibliometric analysis is a method to quantitatively analyze publications, using mathematical and statistical methods (6). Bibliometrics is used to quantify the influence of research and to determine the structure of research fields by means of performance analysis and bibliometric networks. This provides information on the current status of knowledge in a specific area and supports the development of future lines of research (7). Over the years, bibliometric analyses regarding physical exercise and epilepsy, epilepsy and circadian rhythm, and suicide associated with epilepsy have been reported (6, 8, 9); however, a bibliometric analysis of DRE literature has not been published now.

In this study, VOSviewer, R software, and CiteSpace were used to build a network of countries, affiliations, co-authors, co-occurrent keywords, bibliographic coupling analysis, co-citations, and references with citation bursts. We further analyzed the 100 most cited publications in this area between 2011 and 2021 to highlight evidence-based studies in the field. The purpose of our article was to systematically and visually describe and analyze DRE research, as well as to provide researchers with meaningful insight into future directions related to the mechanisms and management of DRE.

## Methods

### Data sources and search strategies

The Web of Science (WoS) core collection Science Citation Index Expanded (SCI-expanded) database (Thomson Reuters Scientific, Philadelphia, PA, USA) was utilized to conduct bibliometric analysis. To avoid deviations, two researchers (Xiao-Jun Ni and Han Zhong) independently performed the literature search on a single day (February 10, 2022) and determined the eligibility of potential articles. The publication period was set between 2011 and 2021. The search strategy was derived from a systematic review (10) (Appendix 1). Among the broad range of publication types, only articles written in English were retained. The original data were extracted in text format from the database previously mentioned. The recorded data included details of the author(s), title, abstract, keywords, sources, affiliations, citations, references, country/region, and publication year of each article.

### Bibliometric analysis

The online analysis function of the WoS core database was used to extract basic information on the included publications, including annual, national, institutional, and individual numbers of publications (NOP), the number of citations not including self-citations (NOC), and the top 100 cited publications. The H-index is an integrated indicator used to assess the academic achievements of a researcher, a journal, an institution, or a nation or region and predict future scientific contribution, unifying productivity and impact by finding the appropriate threshold that connects NOP with NOC. Journal impact factors were obtained from the 2021 Journal Citation Reports. The Bibliometrix package (version 3.0) in R software (version 4.1.2, The R Foundation, Vienna, Austria) was used to describe the relationship networks between countries (11). Bibliometric network maps were constructed using VOSviewer (version 1.6.17, Leiden University, Netherlands) based on co-authorship, co-occurrence, bibliographic coupling, and co-citation analysis. Each circle in the VOSviewer maps represented a different element (author, country/region, journal, reference, and keyword occurrence), while the circle size represents the number of elements. The strength of the link is represented by the thickness of the line. The main topic, sub-topic, and type of research of the 100 most cited articles in this field were determined by reading the full text. The connections among the main topic, sub-topic, and type of research in the 100 most cited articles were visualized using Origin 2021 software (OriginLab Corporation, Northampton, MA, USA). CiteSpace is an analytics tool extensively used to visualize and analyze structural and temporal modes in the scientific literature. In this study, CiteSpace (version 5.8. R3, Drexel University, Philadelphia, USA) was used to generate visualizations of terms and references with citation bursts.

## Results

### Overview of DRE publications

**Figure 1:**
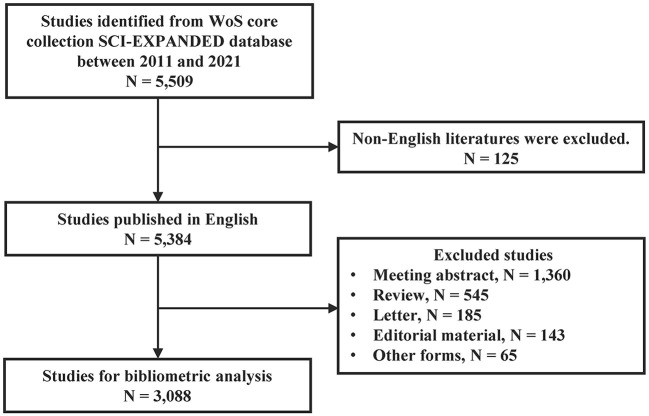
Flow diagram of included publications. WoS, Web of Science.

**Figure 2:**
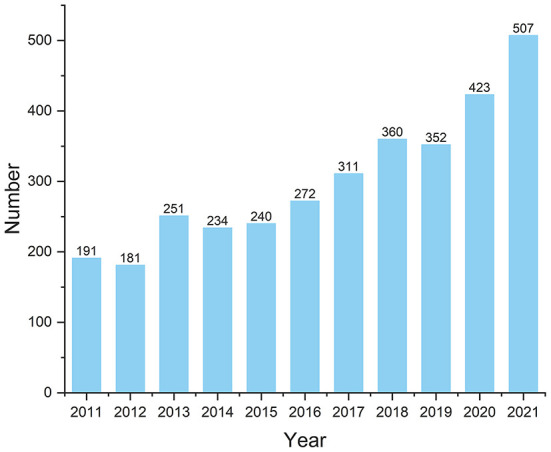
Number of publications by year (2011–2021).

Based on the search strategy, 5,509 publications on DRE were identified between 2011 and 2021. We excluded 125 publications not published in English. A total of 2,296 publications, including 1,360 meeting abstracts, 545 review articles, 185 letters, 143 editorial materials, and 65 other forms, were also removed because of non-pre-defined article types. Ultimately, 3,088 original studies were retained in quantitative analysis. Figure 1 shows a flowchart of the literature search process. The total NOC for the included publications was 42,307, and the mean NOC per publication was 15.68. The H-index of all the papers was 80. The number of annual publications gradually increased from 191 to 507 between 2011 and 2021 (Figure 2).

### Countries or regions, funding agencies, affiliations, and authors

**Figure 3:**
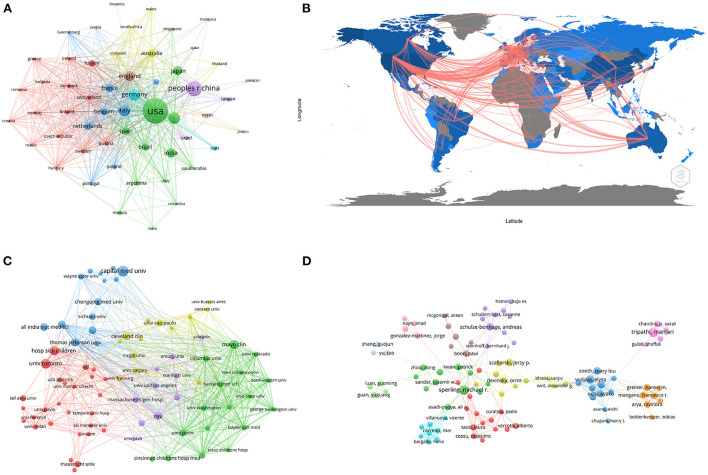
The network map of countries/regions, affiliations, and authors. **(A)** Countries/regions collaboration map based on VOSviewer. **(B)** Countries/regions collaboration map based on R software. **(C)** The cooperation relationship of affiliations. **(D)** The cooperation relationship of authors. Each circle in the VOSviewer maps represented a different element, and the circle size represents the frequency of element occurrences. The strength of the link is represented by the thickness of the line.

A total of 90 countries/regions contributed to DRE research. Table 1 lists the 10 most productive countries/regions. The USA produced the most papers (964; 31.22%) that were cited 22,197 times, accounting for 52.47% of total citations. The USA additionally had the highest H-index (66) and average citation times (24.44) and was thus found to be the most influential country in this field. China had the second highest number of publications (384; 12.44%) with lower average citation times (10.12) and H-index (25). European countries/regions accounted for half of the top 10 high-producing countries/regions, with four of them having an H-index >30, namely Germany, Italy, England, and France. Among Asian countries, in addition to China, India and Japan have also published numerous papers in this field. Strong collaboration existed among these leading countries (Figures 3A,B). The number of collaborators with the USA was 49, and the total link strength was 596, of which the main partners were Canada, Germany, England, Italy, and Belgium. The USA had a greater number of interactions with other countries/regions.

**Table 1:** The top 10 productive countries/regions.

**Rank**	**Country/**	**NOP**	**NOP/total**	**NOC**	**H-index**
	**region**		**publications**		
1	USA	964	31.22	22,197	66
2	China	384	12.44	3,702	25
3	Canada	238	7.71	4,164	31
4	Germany	237	7.67	5,336	39
5	Italy	219	7.09	4,178	32
6	England	173	5.60	3,962	34
7	India	158	5.12	1,368	19
8	Japan	148	4.79	1,338	18
9	France	131	4.24	4,055	32
10	Spain	113	3.66	2,094	23

NOP, the number of publications; NOC, the number of citations not including self-citations.

Table 2 list the top 10 funding agencies. The National Institutes of Health (230), National Natural Science Foundation of China (162), National Institute of Neurological Disorders and Stroke (124), UCB Pharma SA (100), and European Commission (83) were the top five funding agencies.

**Table 2:** The top 10 funding agencies.

**Rank**	**Funding agency**	**Country/region**	**NOP**	**NOC**	**H-index**
1	National Institutes of Health (NIH)	USA	230	5,726	38
2	National Natural Science Foundation of China (NSFC)	China	162	1,356	18
3	National Institutes of Neurological Disorders Stroke (NINDS)	USA	124	4,215	33
4	UCB Pharma SA	Belgium	100	3,795	30
5	European commission	European	83	1,847	26
6	Eisai Co. Ltd.	Japan	77	3,744	27
7	Canada Institutes of health research (CIHR)	Canada	48	1,538	19
8	Pfizer	USA	48	2,276	20
9	GlaxoSmithKline	UK	42	1,904	23
10	Ministry of education culture sports science and technology	Japan	41	282	11

NOP, the number of publications; NOC, the number of citations not including self-citations.

The top 10 most productive affiliations are shown in Table 3. Among them, there were four affiliations from the USA, two from Canada, two from the UK, one from China, and one from France. The University of Toronto had the most productive affiliation, followed by the University of London and Capital Medical University. Harvard University had the highest H-index (27). A co-authorship map of the affiliations is shown in Figure 3C. The University of Toronto had built a large cooperation network, the main collaborator of which was the Hospital for Sick Children.

**Table 3:** The top 10 affiliations.

**Rank**	**Affiliation**	**Country/region**	**NOP**	**NOC**	**H-index**
1	University of Toronto	Canada	114	1,849	24
2	University of London	England	93	2,566	26
3	Capital Medical University	China	92	713	15
4	Harvard University	USA	89	3,839	27
5	Hospital for Sick Children (Sickkids)	Canada	84	1,024	20
6	University of California System	USA	81	2,945	25
7	Institut National De La Sante ET DE LA Recherche Medicale (Inserm)	France	79	1,921	25
8	University College London	England	76	2,273	24
9	Mayo Clinic	USA	70	1,932	20
10	Cleveland Clinic Foundation	USA	61	2,116	21

NOP, the number of publications; NOC, the number of citations not including self-citations.

Between 2011 and 2021, a total of 14,147 authors have published relevant research papers. The 11 most productive authors are shown in Table 4. They contributed 299 publications, accounting for 9.68% of the total number of papers. The top five productive authors were Tripathi M from the All India Institute of Medical Sciences, Sperling MR from Thomas Jefferson University, Wang XF from Chongqing Medical University, Schulze-Bonhage A from University Hospital Freiburg, and Widjaja E from the Hospital for Sick Children. Tripathi M focused on precision surgical treatment of intractable epilepsy, especially neuromodulation; Sperling MR focused on risk factors associated with seizure outcome and antiseizure medication, and Wang XF focused on refractory epilepsy and its various biomarkers. Tripathi M's main collaborators were Chandra PS, Garg A, and Ramanujam B from the All India Institute of Medical Sciences. Most of the top 11 authors were from Canada (3), the USA (3), and India (2). Authors who published more than 10 articles were divided into 15 clusters on the map of the collaboration network (Figure 3D). Major research teams were identified, and these top authors were included.

**Table 4:** The top 11 authors with the most publications.

**Rank**	**Author**	**Country/region**	**NOP**	**NOC**	**H-index**
1	Manjari Tripathi	India	36	469	12
2	Michael R Sperling	USA	34	1,958	16
3	Xue-Feng Wang	China	33	352	11
4	Andreas Schulze-Bonhage	Germany	28	686	15
5	Elysa Widjaja	Canada	28	316	11
6	P Sarat Chandra	India	24	348	8
7	O Carter Snead	Canada	24	431	14
8	Ravindra Arya	USA	23	213	9
9	Josemir W Sander	England	23	298	10
10	Mary Lou Smith	Canada	23	249	11
11	Jerzy P Szaflarski	USA	23	348	11

NOP, the number of publications; NOC, the number of citations not including self-citations.

### Analysis of journals, keywords, bibliographic coupling, and co-cited references

**Figure 4:**
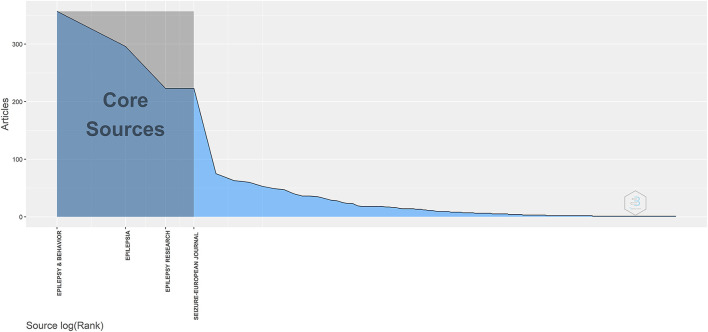
Bradford's Law applied to the sources.

A total of 528 journals had published papers on DRE. Table 5 presents the top 10 most productive journals. Papers published in these journals accounted for 46.86% (1,447/3,088) of publications in the WoS core database. There were four journals with more than 200 publications, of which *Epilepsy* & *Behavior* (357 publications, IF 2021 3.337) ranked first, followed by *Epilepsia* (297 publications, IF 2021 6.740), *Epilepsy Research* (223 publications, IF 2021 2.991), and *Seizure* (223 publications, IF 2021 3.414). Applying Bradford's Law, *Epilepsy* & *Behavior, Epilepsia, Epilepsy Research*, and *Seizure* ranked first among all sources in the field of DRE (Figure 4). Among the top 10 journals, five journals had an impact factor greater than three. *Epilepsia* had the highest H-index value (54).

**Table 5:** The top 10 most active journals.

**Rank**	**Journal**	**NOP**	**NOC**	**H-index**	**IF (2021)**
1	Epilepsy & Behavior	357	4,343	31	3.337
2	Epilepsia	297	9,582	54	6.740
3	Epilepsy Research	223	3,160	29	2.991
4	Seizure	223	3,114	29	3.414
5	Epileptic Disorders	75	670	15	2.333
6	Frontiers in Neurology	63	377	12	4.086
7	Journal of Child Neurology	60	662	14	2.363
8	Brain and Development	53	475	13	2.272
9	Pediatric Neurology	49	607	14	4.210
10	Journal of Neurosurgery Pediatrics	47	537	13	2.713

NOP, the number of publications; NOC, the number of citations not including self-citations; IF, impact factor.

**Figure 5:**
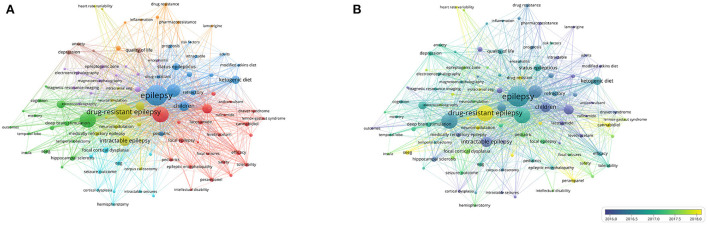
The network mapping on keywords. **(A)** Network visualization map. The 99 keywords were divided into eight clusters with different colors. **(B)** Overlay Visualization map. Keywords in yellow appeared later than that in blue.

Keywords provided by the authors of the publication with an occurrence of over 15 times in the WoS core database were included in the analysis. Among 5,287 keywords, 99 met these criteria. The three most frequent keywords were “epilepsy” (total link strength of 1,194), “epilepsy surgery” (total link strength of 813), and “refractory epilepsy” (total link strength of 609). “epilepsy” had a strong link to “seizure”, “epilepsy surgery”, “children”, “antiepileptic drug” and “vagus nerve stimulation”. Additionally, “drug-resistant epilepsy”, “seizure”, “children”, “antiepileptic drug”, “vagus nerve stimulation”, “ketogenic diet”, “intractable epilepsy” and “status epilepticus” were other keywords with a total link strength greater than 200 (Figures 5A,B). As shown in Figure 5A, the keywords were classified into eight clusters of distinct colors. Clusters 1 (red) and 7 (orange) were primarily recent antiseizure medications, which included “perampanel”, “brivaracetam”, “cannabidiol”, “lacosamide”, “lamotrigine”, “safety” and “tolerability”. Clusters 2 (green), 5(purple), and 6 (cyan) focused on epilepsy surgery, particularly presurgical evaluation and screening. Cluster 3 (blue) was about dietary treatment, including the ketogenic and modified Atkins diets. Cluster 4 (yellow) focused on neuromodulation such as responsive neurostimulation and vagus nerve stimulation. Cluster 8 (brown) was about quality of life, mainly mental health. In Figure 5B, the keywords were divided into various colors based on the average publication year (APY). The most recent keywords were “responsive neurostimulation” (cluster 4, APY 2019.79), “cannabidiol” (cluster 1, APY 2019.10), “neuromodulation” (cluster 4, APY 2019.00), “seeg” (cluster 3, APY 2018.51), and “perampanel” (cluster 1, APY 2018.13).

Bibliographic coupling analysis weighs links between papers that have cited the same literature. Supplementary Figures 1A,B demonstrate the bibliographic coupling networks for documents and journals. Documents with the 500 greatest total link strength were selected for document analysis. The colored clusters focused on different fields. Four clusters were generated for the analysis. Cluster 1 (red) included 216 items, which mainly pertained to diagnosis, outcome prediction, risk factors, and quality of life of DRE patients. Cluster 2 (green) mainly focused on epilepsy surgery and responsive neurostimulation. Cluster 3 (blue) was centered on the effectiveness and tolerability of multiple ASMs. The topic of cluster 4 (in yellow) was clinical studies on vagus nerve stimulation. For the journal analysis, six clusters were generated, the largest of which included 30 items. *Epilepsy* & *Behavior* was the most representative journal.

The co-citation network emphasizes research themes closely associated with a specific field. Co-citations represent how frequently two papers are cited together by other papers and may be considered as a knowledge base for a specific field (12). Given the number of cited references, the minimum number of citations of a reference was set to 25. Of the 53,428 references cited in the retained papers, 146 were identified for co-citation analysis. The co-citation networks for documents and journals are presented in Supplementary Figures 1C,D, respectively. The co-cited references were classified into seven clusters. The top three clusters focused on surgical therapy (red), including preoperative risk assessment and surgical techniques, the definition of DRE epilepsy (green), and vagus nerve stimulation (blue). When two journals are cited simultaneously in one or more publications, they are regarded to have a co-citation relationship. There were 7,344 co-cited journals, and 5 journals had over 3,000 co-citations. *Epilepsia* had the highest number of co-citations (16,069), followed by *Neurology* (5,429), *Epilepsy* & *Behavior* (4,519), *Epilepsy Research* (3,494), and *Seizure* (*n* = 3,081). Journals with over 200 co-citations were used to develop the co-citation network. As illustrated in Supplementary Figure 1D, *Epilepsia* had an active co-citation relationship with *Neurology, Epilepsy* & *Behavior, Epilepsy Research*, and *Brain*.

### Top 100 most cited publications

**Figure 6:**
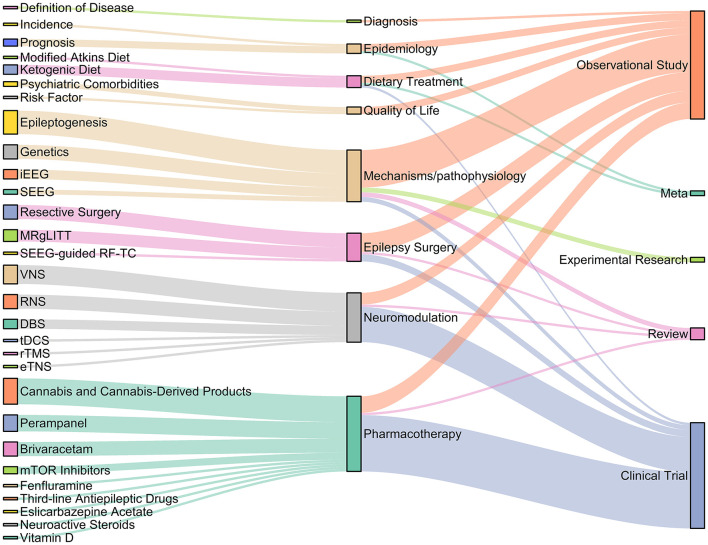
Article types and topics of the 100 most influential publications. DBS, deep brain stimulation; RNS, responsive neurostimulation; VNS, vagus nerve stimulation; rTMS, repetitive transcranial magnetic stimulation; tDCS, Transcranial Direct Current Stimulation; eTNS, external trigeminal nerve stimulation; MRgLITT, MR-guided laser interstitial thermal therapy; iEEG, intracranial EEG; SEEG, stereoelectroencephalography; SEEG-guided RF-TC, Stereoelectroencephalography-guided radiofrequency thermocoagulation.

The top 100 most cited publications regarding DRE are shown in Supplementary Table 1. Many articles (39) on DRE were published in *Epilepsia*, an official journal of the International League Against Epilepsy (ILAE). Relationships between the topics and subtopics of the aforementioned articles were established (Figure 6). Among the top 100 most impactful articles, there were 91 clinical studies, including 45 clinical trials and 46 observational studies. In addition, five reviews, two meta-analyses, and two experimental studies were included. Pharmacotherapy was the most studied topic (32), followed by disease mechanism/pathophysiology (22), neuromodulation (21), and surgical therapy (11). Cannabis and cannabis-derived products (11) were the most studied subtopics, followed by epileptogenesis (10), vagus nerve stimulation (8), and perampanel (7).

The number of most cited articles on pharmacotherapy peaked in 2013 and decreased rapidly over the next few years. The number of articles on disease mechanisms/pathophysiology remained stable from 2011 to 2016 and decreased between 2017 and 2019.

### Terms and references with citation bursts

**Figure 7:**
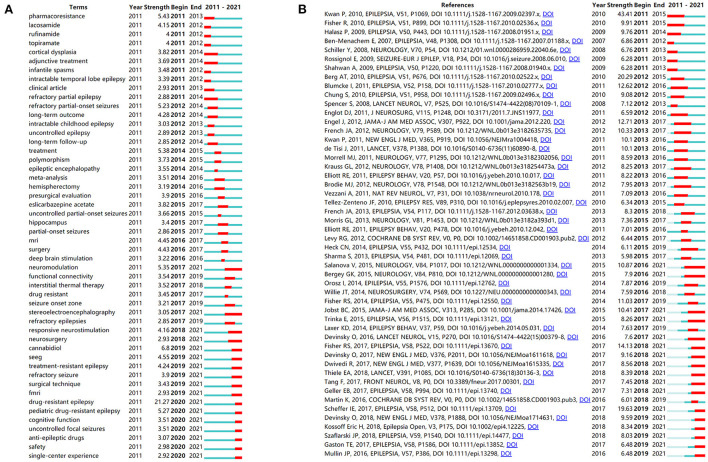
Burst detection **(A)** Top 50 terms with the strongest citation bursts. **(B)** Top 50 references with the strongest citation bursts.

Citation burst detection can reveal research trends and novel topics that are rapidly gaining attention in a specific area. Figure 7A shows the top 50 terms with the most substantial citation bursts. The terms were sourced from titles, author keywords, and keywords plus. The blue line represents the period from 2011 to 2021, whereas the red line represents the time interval of citation bursts. We found that the burst of terms related to ASMs constantly changed. In the earlier period from 2011 to 2012, the burst terms were lacosamide, rufinamide, and topiramate, followed by eslicarbazepine acetate during the mid-term period of 2015–2017, and cannabidiol in the last period of 2019–2021. Terms related to surgery or neuromodulation were always topical, especially in later periods, which chronologically included hemispherectomy, presurgical evaluation, surgery, deep brain stimulation, neuromodulation, interstitial thermal therapy, seizure onset zone, stereoelectroencephalography, responsive neurostimulation, neurosurgery, and surgical technique. Figure 7B shows the top 50 references with the highest number of citation bursts. The strongest burst (strength = 43.41) was the publication entitled “Definition of drug resistant epilepsy: consensus proposal by the *ad-hoc* task force of the ILAE Commission on Therapeutic Strategies” published in *Epilepsia* by Kwan et al., with the burst lasting from 2011 to 2015. This was followed by “Revised terminology and concepts for organization of seizures and epilepsies: report of the ILAE Commission on Classification and Terminology, 2005–2009” (strength = 20.29), and “ILAE classification of the epilepsies: Position paper of the ILAE Commission for Classification and Terminology” (strength = 19.63). These cited papers are landmark documents in this field, which effectively standardized the definition and classification of epilepsy, and provided a foundation for subsequent studies.

## Discussion

In the current study, we conducted a bibliometric analysis to provide an overview of developmental trends and hotspots in research on DRE from the SCI-expanded database, using a variety of bibliometric tools. Due to many leading affiliations, funding agencies, and researchers, the USA made the most important contribution to this particular research field. As the predominant research teams in Asian countries, China, India, and Japan must make more efforts to conduct innovative research to increase the impact. Regarding journals, *Epilepsia* is the most influential journal in this field owing to the number and importance of publications.

As seen in the analysis of the top 100 most cited publications, the most influential studies were clinical studies. These studies analyzed the diagnosis of DRE, disease characteristics and screening, and multiple treatments such as medication, surgery, neuromodulation, and dietary therapies.

For a long time, the definition of DRE was not unified and potentially conflicting, varying among studies. The study with the highest number of co-citations and the strongest citation bursts was published in 2010 by the ILAE, which delivered a formal consensus on the definition of DRE (3). Only one of the 100 most cited articles evaluated the reliability and validity of the ILAE definitions compared to three other definitions (Berg, Kwan, and Brodie, Camfield, and Camfield) in a clinical setting (13). The ILAE definition received similar acclaim from several subsequent studies (14, 15).

Although DRE patients do not receive two medication regimens, trying other ASMs remains the primary treatment option. From 2011 to 2021, a series of clinical trials on newer third-generation ASMs have been published. Perampanel was approved for treating partial-onset seizures in 2012 and primary generalized tonic clinic seizures in 2016. A pooled analysis demonstrated a significant reduction in seizure frequency (16). Brivaracetam is a modulator of the presynaptic release machinery SV2A closely related to levetiracetam, which was approved in 2016 for treating partial-onset seizures in patients aged 16 years and older. In three phase III trials, adjunctive brivaracetam (50–200 mg/day) showed statistically significant reductions in seizure frequency and was well-tolerated (17–20). Eslicarbazepine acetate was confirmed to be effective and well-tolerated; regardless it was administered as adjunctive therapy or monotherapy (21). Tremendous progress has been made in identifying the genetic causes of epilepsy and investigating the underlying molecular mechanisms, which helps identify potential therapeutic targets and achieve precision medicine (22). Everolimus is a mammalian target of rapamycin inhibitor, which was investigated as an adjunctive therapy to treat focal seizures associated with tuberous sclerosis complex (23). Cannabinoids, mainly cannabidiol, are a potential treatment for patients with Dravet syndrome, Lennox-Gastaut syndrome, tuberous sclerosis complex, and febrile infection-related epilepsy syndrome (24–28). In 2018, a purified form of cannabidiol (Epidiolex) was approved for treating patients aged 2 years and older with rare epilepsy syndromes (e.g., Lennox-Gastaut syndrome or Dravet syndrome). Fenfluramine, a serotonin-releasing agent, showed efficacy and safety in treating Dravet syndrome (27) and was approved in 2020 to prevent seizures in pediatric patients with Dravet syndrome.

The current choice of ASMs can be described as empirical and based primarily on the patient's demographic profile, seizure type, comorbidity, concomitant medication, and adverse drug reaction. Exploration of pharmacogenomics in epilepsy may help to predict drug responses and improve effectiveness and tolerability. Genetic variations of drug transporters may be associated with a higher risk of DRE (29). ATP-binding cassette subfamily B member 1 (ABCB1) polymorphisms affect ASM brain penetration and efficiency. A meta-analysis showed a relationship between ABCB1 C3435T polymorphism and poor treatment response (30). Given multiple factors are involved in DRE, pharmacokinetic factors may not be enough to explain the differences in drug response. Whole-genome sequencing technology, genome-wide association studies, and polygenic risk score (PRS) open a broader window to understand the mechanisms of drug resistance and specific ASM responses. Several candidate rare genetic variants were found in the exome-based research of patients with non-familial non-acquired focal epilepsy and DEPDC5 was regarded as a potential risk factor for drug resistance (31). For the specific drug response, common genetic variants were not significantly associated with common ASMs in a study containing 3,649 individuals with focal epilepsy or generalized genetic epilepsy (32). However, an exome-based study targeting rare genetic variants revealed an increased burden of damaging variants in gene groups associated with pharmacokinetics or targeting in patients resistant to valproic acid or levetiracetam (33). These findings may hold promise for patients with epilepsy, allowing early prediction of drug efficacy and optimization of treatment strategies.

To treat DRE, non-AED treatment options may be considered, including epilepsy surgery, neurostimulation, and dietary therapy. Among these, epilepsy surgery carries the potential for long-term seizure control; however, only some patients with drug-resistant focal epilepsy are good surgical candidates. An MRI lesion that can be completely resected is a significant prognostic factor for long-term postoperative seizure control (1). A meta-analysis suggested that the median proportion of long-term seizure-free patients with temporal lobe surgery was 66% (34), which was similar to short-term outcomes (35). Short disease duration was correlated with better postoperative seizure control for intractable frontal lobe epilepsy, whereas left-sided resections and acute seizures after surgery were poor prognostic indicators (35). In children, epilepsy surgery significantly increased the rate of freedom from seizures and improved quality of life (36). However, conventional surgical procedures are associated with high inherent risks, such as visual field deficits, decreased memory, stroke, hemorrhage, and infection (37). In recent times, new techniques with minimal injury have been developed to avoid making a craniotomy. These techniques include radiofrequency ablation (RF-TC) and laser interstitial thermal therapy (LITT). Due to the emergence of stereo electroencephalography (SEEG) recording of ictal activity to guide RF thermocoagulation, RF-TC is considered a palliative treatment for patients who are not eligible for open surgery. A meta-analysis of six retrospective studies revealed that seizure-free and responder rates 1 year after SEEG-guided RF-TC were 23 and 58%, respectively. Subgroup analyses showed that the greatest efficacy was found in patients with periventricular nodular heterotopia (38% seizure-free and 81% responders) and the lowest in patients with normal MRI (11% seizure-free and 41% responders) (38). A study of 162 patients with SEEG-guided RF-TC found that 67% of responders at 2 months, 48% at 1 year, and 58% of responders maintained their status during a 10-year follow-up (39). Current data have affirmed the superiority of SEEG-guided RF-TC in terms of safety. However, differences exist in underlying lesions and management strategies across centers, which affect the clinical efficacy (40). LITT utilizes laser thermal ablation *via* a fiber probe inserted through a twist drill hole with the advantage of ablating difficult-to-reach epileptogenic regions. A multicenter cohort study including 234 patients who underwent LITT for mesial temporal lobe epilepsy demonstrated that Engel I outcome was achieved in 58% of patients at both 1 and 2 years after LITT. A single-center study including pediatric patients with drug-resistant lesional epilepsy showed that Engel class I outcome was achieved in 41% patients (41). In a quantitative analysis of novel “minimally invasive” approaches, the pooled seizure-free rate per person-year was 0.59 with LITT and 0.38 with RF-TC, which were not as efficacious as open surgery (42). Nevertheless, LITT and RF-TC remain surgical options, even as a first-line treatment for certain epilepsy indications, while more high-quality evidence is needed.

Neuromodulation is an alternative therapy for patients who fail to respond to ASMs and are not suitable for open surgery; this has become a research hotspot. Neuromodulation includes invasive therapies such as vagus nerve stimulation (VNS), deep brain stimulation (DBS), responsive neurostimulation (RNS), and non-invasive methods including transcutaneous VNS and transcranial stimulation. Neuromodulation is considered a palliative treatment, that only some patients might benefit from for longer than 12 months (43). VNS was the first neurostimulation device approved for epilepsy. A retrospective study of 436 consecutive adults and children who underwent VNS demonstrated that 63.75% of the patients achieved ≥ 50% seizure reduction during the treatment period ranging from 10 days to 11 years (44). Two large systematic reviews found that the prevalence of 50% responder rate and seizure freedom at the last follow-up (mean 2.54 years) were 56.4 and 11.6%, respectively, in children and 63 and 8.2%, respectively, in adults (45, 46). VNS has also proven to improve the quality of life in patients with DRE (47).

The closed-loop responsive neurostimulation system was designed to abort possible impending seizures by monitoring the electrocorticogram at the seizure focus and delivering short bursts of high-frequency electrical stimulation. The acute and sustained efficacy and safety of the RNS system were confirmed in a long-term study (mean 5.4 years). The median percent seizure reduction rate ranged from 44 to 66% over 1–6 years post-implant (48). A 9-year follow-up study of patients continuing RNS revealed RNS system progressively increased the median reduction in seizure frequency and 50% responder rate, which reached 75 and 73%, respectively (49). The challenge of neuromodulation lies in selecting the most suitable neuromodulation techniques and targets according to the type of epilepsy. In addition, a flowchart of neuromodulation and other treatments for drug-resistant focal epilepsy has been developed, which provides a route for treatment from the beginning of drug resistance (43).

Ketogenic dietary therapy has been used since the 1920's, mainly in pediatric patients with severe and refractory epilepsy. In the past decade, owing to the availability of broader dietary options and the expansion of the target population, the ketogenic diet has received renewed attention. The efficacy of the ketogenic diet has been well-established in children with various seizure types and epileptic syndromes. Children given ketogenic diets may be up to six times more likely to achieve a 50% or greater reduction in seizure frequency and up to three times more likely to become seizure-free than children given the usual care (50). Furthermore, a longitudinal study with a long follow-up period (mean follow-up of 9 years) found that 20.5% of children given a ketogenic diet achieved seizure freedom, and 36% of children had a 75–99% decrease in seizures. (51). However, the ketogenic diet is less effective in adult patients with DRE. Two randomized controlled trials compared the effects of ketogenic diets with those undergoing usual care. Both studies reported 0% seizure freedom in the modified Atkins diet group, with >50% seizure reduction in 8 and 35% of patients in the modified Atkins diet group, respectively (52, 53).

Based on the bibliometric analysis of the literature in DRE, we provide a comprehensive and objective insight into the global status and hotspots in this field. However, this study has some limitations. First, only articles written in English from the SCI-expanded database were included. Second, some emerging significant papers and trends may not have been captured due to a low number of citations. Despite these limitations, this study provides insight into research from the last decade in this special field, demonstrating that there is sustained and intense interest in identifying the mechanisms of DRE and developing various antiseizure therapies to improve the outcome of patients with DRE.

## Conclusion

This bibliometric analysis has demonstrated that DRE has always been a hot area of research. The USA is the most influential and productive country, with extensive co-operation with other leading countries. *Epilepsy* & *Behavior, Epilepsia, Epilepsy Research*, and *Seizure* were the most productive journals, of which *Epilepsia* published many papers with several citations. The bibliometric data can make important contributions to our understanding of the advancement of DRE management.

## Data availability statement

The original contributions presented in the study are included in the article/Supplementary material, further inquiries can be directed to the corresponding author/s.

## Author contributions

X-JN and HZ performed the data search and drafted the manuscript. Y-XL performed the software. Z-CG and H-WL revised and approved the final manuscript. All authors made substantial contributions to the conception and design of this study. All authors contributed to the article and approved the submitted version.

## Funding

This work was supported by the Clinical Research Innovation and Cultivation Fund of Ren Ji Hospital (RJPY-LX-008) and Research Project of Drug Clinical Comprehensive Evaluation and Drug Treatment Pathway (SHYXH-ZP-2021-001).

## Conflict of interest

The authors declare that the research was conducted in the absence of any commercial or financial relationships that could be construed as a potential conflict of interest.

## Publisher's note

All claims expressed in this article are solely those of the authors and do not necessarily represent those of their affiliated organizations, or those of the publisher, the editors and the reviewers. Any product that may be evaluated in this article, or claim that may be made by its manufacturer, is not guaranteed or endorsed by the publisher.
