# Supplementary material for: Current trends and hotspots in drug-resistant epilepsy research: Insights from a bibliometric analysis
Source: Front Neurol. 2022 Nov 3;13:1023832. doi: 10.3389/fneur.2022.1023832 (PMC9669477; doi:10.3389/fneur.2022.1023832)
Supplement: Supplementary file 1 [file Data_Sheet_1.docx]

**Appendix1. Web of Science Search Strategy**

(TI=(epileps* OR seizure* OR convulsion*) OR AK=(epileps* OR seizure* OR convulsion*)) AND (TI= ((refractory) OR (intractable) OR (uncontrolled) OR (treatment resistant) OR (medication resistant) OR (drug resistant) OR (pharmacoresistant) OR (pharmaco resistant) OR (treatment failure)) OR AK= ((refractory) OR (intractable) OR (uncontrolled) OR (treatment resistant) OR (medication resistant) OR (drug resistant) OR (pharmacoresistant) OR (pharmaco resistant) OR (treatment failure)))


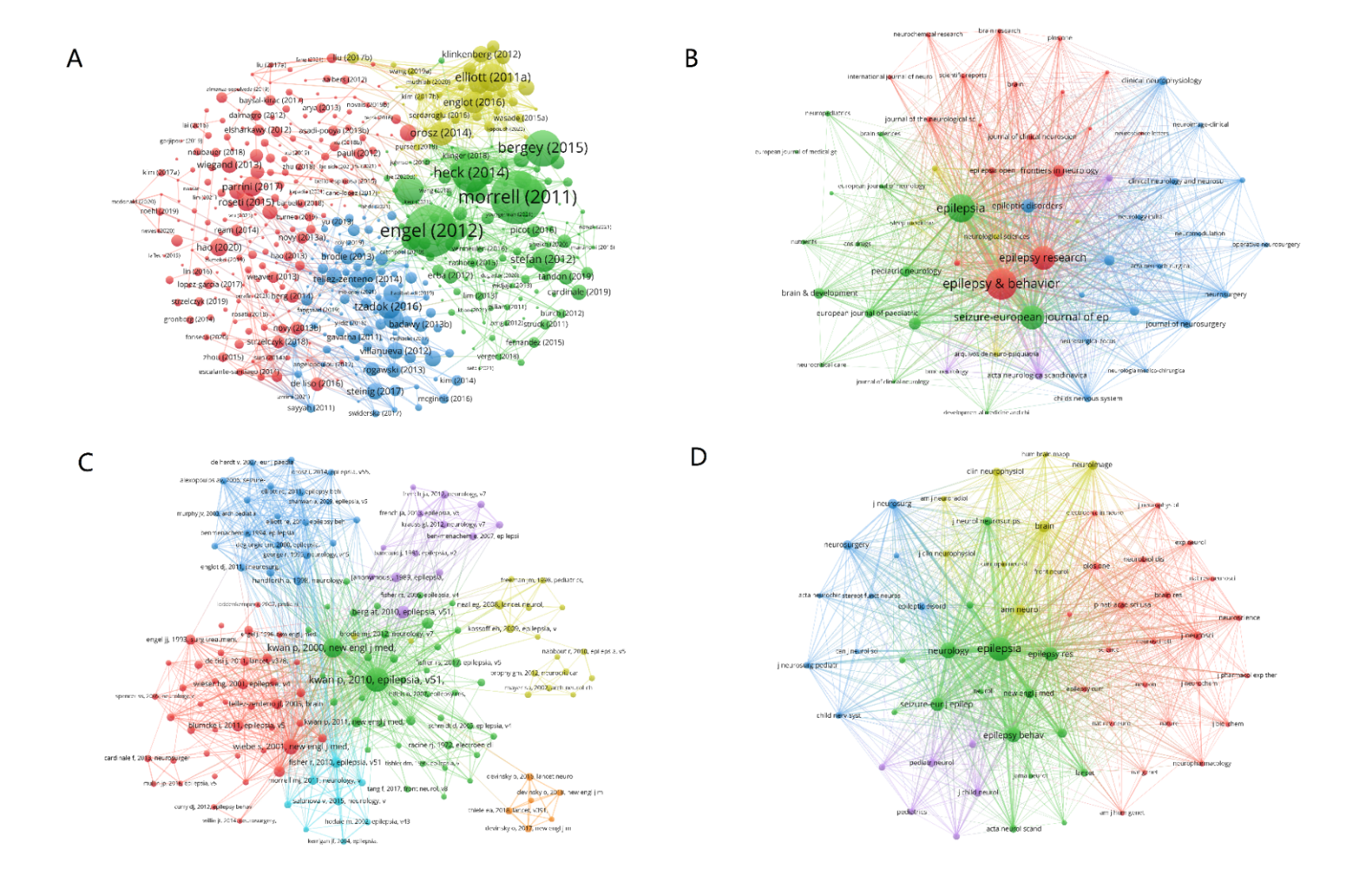


**Figure S1. Bibliometric analysis of the bibliographic coupling and co-citation. (A) Bibliographic coupling map of documents. (B) Bibliographic coupling map of sources. (C) Co-citation map of documents. (D) Co-citation map of sources. The size of the nodes indicates the counts of bibliographic coupling or co-citations and the distance between two nodes represent their correlation.**

**Table S1. The top 100 most cited publications**

| **Rank** | **Article Title** | **Journal** | **Publication Year** | **Times Cited** | **DOI** |
| --- | --- | --- | --- | --- | --- |
| 1 | Responsive cortical stimulation for the treatment of medically intractable partial epilepsy | Neurology | 2011 | 663 | 10.1212/WNL.0b013e3182302056 |
| 2 | Early Surgical Therapy for Drug-Resistant Temporal Lobe Epilepsy A Randomized Trial | JAMA-J. Am. Med. Assoc. | 2012 | 661 | 10.1001/jama.2012.220 |
| 3 | Trial of Cannabidiol for Drug-Resistant Seizures in the Dravet Syndrome | N. Engl. J. Med. | 2017 | 652 | 10.1056/NEJMoa1611618 |
| 4 | Cannabidiol in patients with treatment-resistant epilepsy: an open-label interventional trial | Lancet Neurol. | 2016 | 473 | 10.1016/S1474-4422(15)00379-8 |
| 5 | Prediction of seizure likelihood with a long-term, implanted seizure advisory system in patients with drug-resistant epilepsy: a first-in-man study | Lancet Neurol. | 2013 | 441 | 10.1016/S1474-4422(13)70075-9 |
| 6 | Long-term efficacy and safety of thalamic stimulation for drug-resistant partial epilepsy | Neurology | 2015 | 344 | 10.1212/WNL.0000000000001334 |
| 7 | Two-year seizure reduction in adults with medically intractable partial onset epilepsy treated with responsive neurostimulation: Final results of the RNS System Pivotal trial | Epilepsia | 2014 | 331 | 10.1111/epi.12534 |
| 8 | Adjunctive everolimus therapy for treatment-resistant focal-onset seizures associated with tuberous sclerosis (EXIST-3): a phase 3, randomised, double-blind, placebo-controlled study | Lancet | 2016 | 333 | 10.1016/S0140-6736(16)31419-2 |
| 9 | Encephalitis with refractory seizures, status epilepticus, and antibodies to the GABA(A) receptor: a case series, characterisation of the antigen, and analysis of the effects of antibodies | Lancet Neurol. | 2014 | 335 | 10.1016/S1474-4422(13)70299-0 |
| 10 | Adjunctive perampanel for refractory partial-onset seizures Randomized phase III study 304 | Neurology | 2012 | 316 | 10.1212/WNL.0b013e3182635735 |
| 11 | Long-term treatment with responsive brain stimulation in adults with refractory partial seizures | Neurology | 2015 | 310 | 10.1212/WNL.0000000000001280 |
| 12 | Randomized phase III study 306 Adjunctive perampanel for refractory partial-onset seizures | Neurology | 2012 | 288 | 10.1212/WNL.0b013e318254473a |
| 13 | Brain somatic mutations in MTOR cause focal cortical dysplasia type II leading to intractable epilepsy | Nat. Med. | 2015 | 279 | 10.1038/nm.3824 |
| 14 | Drug-drug interaction between clobazam and cannabidiol in children with refractory epilepsy | Epilepsia | 2015 | 262 | 10.1111/epi.13060 |
| 15 | Evaluation of adjunctive perampanel in patients with refractory partial-onset seizures: Results of randomized global phase III study 305 | Epilepsia | 2013 | 262 | 10.1111/j.1528-1167.2012.03638.x |
| 16 | Everolimus Treatment of Refractory Epilepsy in Tuberous Sclerosis Complex | Ann. Neurol. | 2013 | 258 | 10.1002/ana.23960 |
| 17 | Report of a parent survey of cannabidiol-enriched cannabis use in pediatric treatment-resistant epilepsy | Epilepsy Behav. | 2013 | 218 | 10.1016/j.yebeh.2013.08.037 |
| 18 | Surgery for Drug-Resistant Epilepsy in Children | N. Engl. J. Med. | 2017 | 206 | 10.1056/NEJMoa1615335 |
| 19 | Interleukin-1 beta Biosynthesis Inhibition Reduces Acute Seizures and Drug Resistant Chronic Epileptic Activity in Mice | Neurotherapeutics | 2011 | 204 | 10.1007/s13311-011-0039-z |
| 20 | Risk of sudden unexpected death in epilepsy in patients given adjunctive antiepileptic treatment for refractory seizures: a meta-analysis of placebo-controlled randomised trials | Lancet Neurol. | 2011 | 195 | 10.1016/S1474-4422(11)70193-4 |
| 21 | Vagus nerve stimulation in 436 consecutive patients with treatment-resistant epilepsy: Long-term outcomes and predictors of response | Epilepsy Behav. | 2011 | 194 | 10.1016/j.yebeh.2010.10.017 |
| 22 | Determinants of health-related quality of life in pharmacoresistant epilepsy: Results from a large multicenter study of consecutively enrolled patients using validated quantitative assessments | Epilepsia | 2011 | 182 | 10.1111/j.1528-1167.2011.03325.x |
| 23 | Laser interstitial thermal therapy for medically intractable mesial temporal lobe epilepsy | Epilepsia | 2016 | 176 | 10.1111/epi.13284 |
| 24 | Efficacy and safety of adjunctive perampanel for the treatment of refractory partial seizures: A pooled analysis of three phase III studies | Epilepsia | 2013 | 174 | 10.1111/epi.12212 |
| 25 | Brivaracetam as adjunctive treatment for uncontrolled partial epilepsy in adults: A phase III randomized, double-blind, placebo-controlled trial | Epilepsia | 2014 | 170 | 10.1111/epi.12433 |
| 26 | Febrile infection-related epilepsy syndrome (FIRES): Pathogenesis, treatment, and outcome A multicenter study on 77 children | Epilepsia | 2011 | 175 | 10.1111/j.1528-1167.2011.03250.x |
| 27 | Stereoelectroencephalography in the difficult to localize refractory focal epilepsy: Early experience from a North American epilepsy center | Epilepsia | 2013 | 161 | 10.1111/j.1528-1167.2012.03672.x |
| 28 | Adjunctive brivaracetam in adults with uncontrolled focal epilepsy: Results from a double-blind, randomized, placebo-controlled trial | Epilepsia | 2014 | 157 | 10.1111/epi.12432 |
| 29 | Parental reporting of response to oral cannabis extracts for treatment of refractory epilepsy | Epilepsy Behav. | 2015 | 148 | 10.1016/j.yebeh.2015.02.043 |
| 30 | A randomized, double-blind, placebo-controlled, multicenter, parallel-group study to evaluate the efficacy and safety of adjunctive brivaracetam in adult patients with uncontrolled partial-onset seizures | Epilepsia | 2015 | 146 | 10.1111/epi.13212 |
| 31 | The case for medical marijuana in epilepsy | Epilepsia | 2014 | 143 | 10.1111/epi.12610 |
| 32 | HIPPOCAMPAL TISSUE OF PATIENTS WITH REFRACTORY TEMPORAL LOBE EPILEPSY IS ASSOCIATED WITH ASTROCYTE ACTIVATION, INFLAMMATION, AND ALTERED EXPRESSION OF CHANNELS AND RECEPTORS | Neuroscience | 2012 | 138 | 10.1016/j.neuroscience.2012.06.002 |
| 33 | Vagus nerve stimulation for drug-resistant epilepsy: A European long-term study up to 24 months in 347 children | Epilepsia | 2014 | 136 | 10.1111/epi.12762 |
| 34 | Transcutaneous vagus nerve stimulation (t-VNS) in pharmacoresistant epilepsies: A proof of concept trial | Epilepsia | 2012 | 140 | 10.1111/j.1528-1167.2012.03492.x |
| 35 | Stereotactic placement of depth electrodes in medically intractable epilepsy | J. Neurosurg. | 2014 | 128 | 10.3171/2013.11.JNS13635 |
| 36 | Brain-responsive neurostimulation in patients with medically intractable mesial temporal lobe epilepsy | Epilepsia | 2017 | 127 | 10.1111/epi.13740 |
| 37 | CBD-enriched medical cannabis for intractable pediatric epilepsy The current Israeli experience | Seizure | 2016 | 124 | 10.1016/j.seizure.2016.01.004 |
| 38 | High-frequency oscillations, extent of surgical resection, and surgical outcome in drug-resistant focal epilepsy | Epilepsia | 2013 | 126 | 10.1111/epi.12075 |
| 39 | Cannabidiol as a new treatment for drug-resistant epilepsy in tuberous sclerosis complex | Epilepsia | 2016 | 118 | 10.1111/epi.13499 |
| 40 | Adjunctive brivaracetam for uncontrolled focal and generalized epilepsies: Results of a phase III, double-blind, randomized, placebo-controlled, flexible-dose trial | Epilepsia | 2014 | 121 | 10.1111/epi.12391 |
| 41 | Hyperphosphorylated tau in patients with refractory epilepsy correlates with cognitive decline: a study of temporal lobe resections | Brain | 2016 | 115 | 10.1093/brain/aww187 |
| 42 | Rates and Predictors of Seizure Freedom With Vagus Nerve Stimulation for Intractable Epilepsy | Neurosurgery | 2016 | 119 | 10.1227/NEU.0000000000001165 |
| 43 | Efficacy of Anti-Inflammatory Therapy in a Model of Acute Seizures and in a Population of Pediatric Drug Resistant Epileptics | PLoS One | 2011 | 113 | 10.1371/journal.pone.0018200 |
| 44 | Transcutaneous Vagus Nerve Stimulation (tVNS) for Treatment of Drug-Resistant Epilepsy: A Randomized, Double-Blind Clinical Trial (cMPsE02) | Brain Stimul. | 2016 | 107 | 10.1016/j.brs.2015.11.003 |
| 45 | A prospective, multicenter study of cardiac-based seizure detection to activate vagus nerve stimulation | Seizure | 2015 | 108 | 10.1016/j.seizure.2015.08.011 |
| 46 | The RNS System: responsive cortical stimulation for the treatment of refractory partial epilepsy | Expert Rev. Med. Devices | 2014 | 106 | 10.1586/17434440.2014.947274 |
| 47 | Crowdsourcing reproducible seizure forecasting in human and canine epilepsy | Brain | 2016 | 105 | 10.1093/brain/aww045 |
| 48 | Improved Outcomes with Earlier Surgery for Intractable Frontal Lobe Epilepsy | Ann. Neurol. | 2013 | 105 | 10.1002/ana.23862 |
| 49 | Brain-responsive neurostimulation in patients with medically intractable seizures arising from eloquent and other neocortical areas | Epilepsia | 2017 | 103 | 10.1111/epi.13739 |
| 50 | Randomized controlled trial of trigeminal nerve stimulation for drug-resistant epilepsy | Neurology | 2013 | 102 | 10.1212/WNL.0b013e318285c11a |
| 51 | Vagus nerve stimulation for children with treatment-resistant epilepsy: a consecutive series of 141 cases Clinical article | J. Neurosurg.-Pediatr. | 2011 | 103 | 10.3171/2011.2.PEDS10505 |
| 52 | The long-term effect of vagus nerve stimulation on quality of life in patients with pharmacoresistant focal epilepsy: The PuLsE (Open Prospective Randomized Long-term Effectiveness) trial | Epilepsia | 2014 | 101 | 10.1111/epi.12611 |
| 53 | Stereotactic laser ablation of epileptogenic periventricular nodular heterotopia | Epilepsy Res. | 2014 | 98 | 10.1016/j.eplepsyres.2014.01.009 |
| 54 | Efficacy and tolerability of adjunctive brivaracetam in adults with uncontrolled partial-onset seizures: A phase IIb, randomized, controlled trial | Epilepsia | 2013 | 98 | 10.1111/j.1528-1167.2012.03598.x |
| 55 | Low-frequency repetitive transcranial magnetic stimulation for the treatment of refractory partial epilepsy: A controlled clinical study | Epilepsia | 2012 | 115 | 10.1111/j.1528-1167.2012.03626.x |
| 56 | Diagnostic Targeted Resequencing in 349 Patients with Drug-Resistant Pediatric Epilepsies Identifies Causative Mutations in 30 Different Genes | Hum. Mutat. | 2017 | 110 | 10.1002/humu.23149 |
| 57 | MR-guided laser interstitial thermal therapy for pediatric drug-resistant lesional epilepsy | Epilepsia | 2015 | 97 | 10.1111/epi.13106 |
| 58 | Remission and relapse in a drug-resistant epilepsy population followed prospectively | Epilepsia | 2011 | 99 | 10.1111/j.1528-1167.2010.02929.x |
| 59 | Third-line antiepileptic therapy and outcome in status epilepticus: The impact of vasopressor use and prolonged mechanical ventilation | Crit. Care Med. | 2012 | 95 | 10.1097/CCM.0b013e3182591ff1 |
| 60 | Long-term cannabidiol treatment in patients with Dravet syndrome: An open-label extension trial | Epilepsia | 2019 | 96 | 10.1111/epi.14628 |
| 61 | Concentration-effect relationships with perampanel in patients with pharmacoresistant partial-onset seizures | Epilepsia | 2013 | 91 | 10.1111/epi.12240 |
| 62 | Long-term safety and treatment effects of cannabidiol in children and adults with treatment-resistant epilepsies: Expanded access program results | Epilepsia | 2018 | 93 | 10.1111/epi.14477 |
| 63 | Differentiation of specific ripple patterns helps to identify epileptogenic areas for surgical procedures | Clin. Neurophysiol. | 2014 | 93 | 10.1016/j.clinph.2013.11.030 |
| 64 | Robot-Assisted Stereotactic Laser Ablation in Medically Intractable Epilepsy: Operative Technique | Oper. Neurosurg. | 2014 | 95 | 10.1227/NEU.0000000000000286 |
| 65 | Costs, length of stay, and mortality of super-refractory status epilepticus: A population-based study from Germany | Epilepsia | 2017 | 90 | 10.1111/epi.13837 |
| 66 | Mammalian Target of Rapamycin Inhibitors for Intractable Epilepsy and Subependymal Giant Cell Astrocytomas in Tuberous Sclerosis Complex | J. Pediatr. | 2014 | 91 | 10.1016/j.jpeds.2013.12.053 |
| 67 | Ictal-onset localization through connectivity analysis of intracranial EEG signals in patients with refractory epilepsy | Epilepsia | 2013 | 87 | 10.1111/epi.12206 |
| 68 | Transcranial Direct Current Stimulation for Treatment of Refractory Childhood Focal Epilepsy | Brain Stimul. | 2013 | 94 | 10.1016/j.brs.2013.01.009 |
| 69 | GLUT1 deficiency syndrome in clinical practice | Epilepsy Res. | 2012 | 90 | 10.1016/j.eplepsyres.2011.02.007 |
| 70 | Depressive and anxiety disorders in epilepsy: Do they differ in their potential to worsen common antiepileptic drug-related adverse events? | Epilepsia | 2012 | 91 | 10.1111/j.1528-1167.2012.03488.x |
| 71 | Use of the modified Atkins diet for treatment of refractory childhood epilepsy: A randomized controlled trial | Epilepsia | 2013 | 89 | 10.1111/epi.12069 |
| 72 | Ketogenic diet poses a significant effect on imbalanced gut microbiota in infants with refractory epilepsy | World J. Gastroenterol. | 2017 | 89 | 10.3748/wjg.v23.i33.6164 |
| 73 | Eslicarbazepine acetate as adjunctive therapy in patients with uncontrolled partial-onset seizures: Results of a phase III, double-blind, randomized, placebo-controlled trial | Epilepsia | 2015 | 84 | 10.1111/epi.12894 |
| 74 | Efficacy of and Patient Compliance with a Ketogenic Diet in Adults with Intractable Epilepsy: A Meta-Analysis | J. Clin. Neurol. | 2015 | 83 | 10.3988/jcn.2015.11.1.26 |
| 75 | Cognitive improvement after long-term electrical stimulation of bilateral anterior thalamic nucleus in refractory epilepsy patients | Seizure | 2012 | 86 | 10.1016/j.seizure.2011.12.003 |
| 76 | Managing drug-resistant epilepsy: challenges and solutions | Neuropsychiatr. Dis. Treat. | 2016 | 85 | 10.2147/NDT.S84852 |
| 77 | GABA(A) currents are decreased by IL-1 beta in epileptogenic tissue of patients with temporal lobe epilepsy: implications for ictogenesis | Neurobiol. Dis. | 2015 | 83 | 10.1016/j.nbd.2015.07.003 |
| 78 | Mutations in QARS, Encoding Glutaminyl-tRNA Synthetase, Cause Progressive Microcephaly, Cerebral-Cerebellar Atrophy, and Intractable Seizures | Am. J. Hum. Genet. | 2014 | 88 | 10.1016/j.ajhg.2014.03.003 |
| 79 | Perampanel, a selective, noncompetitive aleph-amino-3-hydroxy-5-methyl-4-isoxazolepropionic acid receptor antagonist, as adjunctive therapy for refractory partial-onset seizures: Interim results from phase III, extension study 307 | Epilepsia | 2013 | 80 | 10.1111/j.1528-1167.2012.03648.x |
| 80 | Cannabidiol as a Potential Treatment for Febrile Infection-Related Epilepsy Syndrome (FIRES) in the Acute and Chronic Phases | J. Child Neurol. | 2017 | 82 | 10.1177/0883073816669450 |
| 81 | Laser ablation therapy: An alternative treatment for medically resistant mesial temporal lobe epilepsy after age 50 | Epilepsy Behav. | 2015 | 79 | 10.1016/j.yebeh.2015.07.022 |
| 82 | Altered gut microbiome composition in children with refractory epilepsy after ketogenic diet | Epilepsy Res. | 2018 | 81 | 10.1016/j.eplepsyres.2018.06.015 |
| 83 | The pathology of magnetic-resonance-imaging-negative epilepsy | Mod. Pathol. | 2013 | 80 | 10.1038/modpathol.2013.52 |
| 84 | What can we do for people with drug-resistant epilepsy? The 2016 Wartenberg Lecture | Neurology | 2016 | 78 | 10.1212/WNL.0000000000003407 |
| 85 | Long-term safety of perampanel and seizure outcomes in refractory partial-onset seizures and secondarily generalized seizures: Results from phase III extension study 307 | Epilepsia | 2014 | 81 | 10.1111/epi.12643 |
| 86 | Neuroactive steroids for the treatment of status epilepticus | Epilepsia | 2013 | 77 | 10.1111/epi.12289 |
| 87 | Circulating microRNAs are promising novel biomarkers for drug-resistant epilepsy | Sci Rep | 2015 | 76 | 10.1038/srep10201 |
| 88 | Correction of vitamin D deficiency improves seizure control in epilepsy: A pilot study | Epilepsy Behav. | 2012 | 76 | 10.1016/j.yebeh.2012.03.011 |
| 89 | Long-term follow-up of the ketogenic diet for refractory epilepsy: Multicenter Argentinean experience in 216 pediatric patients | Seizure | 2011 | 80 | 10.1016/j.seizure.2011.06.009 |
| 90 | Severe psychological distress among patients with epilepsy during the COVID-19 outbreak in southwest China | Epilepsia | 2020 | 75 | 10.1111/epi.16544 |
| 91 | DNM1L-related mitochondrial fission defect presenting as refractory epilepsy | Eur. J. Hum. Genet. | 2016 | 75 | 10.1038/ejhg.2015.243 |
| 92 | Refractory epilepsy and mitochondrial dysfunction due to GM3 synthase deficiency | Eur. J. Hum. Genet. | 2013 | 72 | 10.1038/ejhg.2012.202 |
| 93 | A validation of the new definition of drug-resistant epilepsy by the International League Against Epilepsy | Epilepsia | 2014 | 73 | 10.1111/epi.12633 |
| 94 | A DECADE OF EXPERIENCE WITH DEEP BRAIN STIMULATION FOR PATIENTS WITH REFRACTORY MEDIAL TEMPORAL LOBE EPILEPSY | Int. J. Neural Syst. | 2013 | 73 | 10.1142/S0129065712500347 |
| 95 | Long-Term Outcome of Anterior Thalamic Nucleus Stimulation for Intractable Epilepsy | Stereotact. Funct. Neurosurg. | 2012 | 72 | 10.1159/000339991 |
| 96 | Postmarketing experience with brivaracetam in the treatment of epilepsies: A multicenter cohort study from Germany | Epilepsia | 2017 | 66 | 10.1111/epi.13768 |
| 97 | Burden of uncontrolled epilepsy in patients requiring an emergency room visit or hospitalization | Neurology | 2012 | 66 | 10.1212/WNL.0b013e318271f77e |
| 98 | Epilepsyecosystem.org: crowd-sourcing reproducible seizure prediction with long-term human intracranial EEG | Brain | 2018 | 65 | 10.1093/brain/awy210 |
| 99 | Low-dose fenfluramine significantly reduces seizure frequency in Dravet syndrome: a prospective study of a new cohort of patients | Eur. J. Neurol. | 2017 | 67 | 10.1111/ene.13195 |
| 100 | Stereo electroencephalography-guided radiofrequency thermocoagulation (SEEG-guided RF-TC) in drug-resistant focal epilepsy: Results from a 10-year experience | Epilepsia | 2017 | 77 | 10.1111/epi.13616 |
